# Draft Genome of the Insect-Parasitic Nematode *Bradynema Listronoti*

**DOI:** 10.2478/jofnem-2022-0047

**Published:** 2022-12-14

**Authors:** Dave T. Ste-Croix, Annie-Ève Gagnon, Benjamin Mimee

**Affiliations:** 1 Agriculture and Agri-Food Canada, Saint-Jean-sur-Richelieu Research and Development Centre, Saint-Jean-sur-Richelieu, QC J3B 3E6, Canada

**Keywords:** Bradynema listronoti, entomoparasitic nematode, genomics, MinION, NovaSeq, whole-genome amplification

## Abstract

*Bradynema listronoti* is an insect-parasitic nematode known to infect the carrot weevil, *Listronotus oregonensis*. We present the first sequence for this species and for any Allantonematidae, produced with a combination of short and long reads. The draft genome of *B. listronoti* is 80.6 Mb in size, assembled in 152 scaffolds.

The genus *Bradynema* (Nematoda: Allantonematidae), first characterized by Strassen in 1892, currently comprises nine species of insect-parasitic nematodes with a diverse set of hosts. In 2007, a novel species, *Bradynema listronoti*, was isolated from the hemocoel of adult carrot weevils, *Listronotus oregonensis* (Coleoptera: Curculionidae), an important pest of horticultural crops (Zeng *et al*., (2007)). It was shown that *B. listronoti* castrates its host by inhibiting the maturation of the reproductive system in females (Gagnon *et al*., (2007) 2019), and its potential as a biological control agent is currently investigated. In comparison to entomopathogenic nematodes, this group has been studied very little, despite its importance in the regulation of insect populations. In this study, we combined short- and long-read sequencing data to assemble a high-quality genome for *B. listronoti*.

*B. listronoti* specimens (strain AAFC-BrLi-01) used in this study were isolated from a colony of infected carrot weevils at the Saint-Jean-sur-Richelieu Research and Development Centre of Agriculture and Agri-Food Canada. This colony was established following the initial isolation of *B. listronoti* by Zeng *et al*. (2007). Genomic DNA was obtained from an individual juvenile (J3) nematode and a pool of J3. For the single nematode, DNA was extracted and amplified using the REPLI-g Advanced DNA Single Cell Kit (Qiagen, Hilden, Germany). To resolve the hyperbranching generated by this whole-genome amplification (WGA) protocol, 5 μg of WGA gDNA was treated with T7 endonuclease, and short fragments were removed using the Circulomics SRE XS Kit (Circulomics, Baltimore, MD). From this size-selected material, two Nanopore MinION libraries were generated, from 1 μg of genomic material each, using the Ligation Sequencing Kit chemistry (SQK-LSK109, Oxford Nanopore Technologies, Oxford, United Kingdom). Short fragments were eliminated using the L Fragment Buffer provided in the kit prior to sequencing on MinION R9.4.1 flow cells. Using the same WGA gDNA, a short-read shotgun sequencing library was prepared using the NEBNext Ultra II DNA Library Preparation Kit and paired-end-sequenced on the Illumina NovaSeq6000 platform (2 × 150 bp) at the McGill University and Génome Québec Innovation Centre. In a second experiment, high-molecular weight (HMW) gDNA was prepared from a pool of ~15,000 J3 nematodes for downstream assembly scaffolding purposes. Genomic DNA was extracted using the Monarch HMW DNA Extraction Kit for Tissue (New England Biolabs, Ipswich, MA) following the manufacturer’s recommendations. The sequencing library was prepared from 1 μg of genomic material using the Ligation Sequencing Kit (Q20+) chemistry (SQK-LSK112) and sequenced on a MinION R10.4 flow cell. Messenger RNA was extracted and amplified from multiple *B. listronoti* stages (egg, J2, J3, and J4) using the method described in Ste-Croix *et al*. (2021) with three biological replicates of each. Sequencing libraries were prepared using the NEBNext Ultra II DNA Library Preparation Kit and paired-end-sequenced on the NovaSeq6000 platform (2 _×_ 150 bp). Genome size was estimated using GenomeScope (Vurture *et al*., 2017) with k-mer frequency distributions derived from single-nematode whole-genome shotgun sequencing data.

The different sequencing technologies generated 33 Gb of long-read sequences alongside 5.3 Gb of Q20+ long-read sequences derived from HMW gDNA, and 20.7 Gb of paired-end short reads. Additionally, mRNA sequencing generated 706 M reads for an estimated 210 Gb of data. For long- and short-read sequencing data, sequences were trimmed and filtered based on quality using Guppy (v 6.0.6) and Fastp (v 0.20.1 – Chen *et al*., 2018), respectively. The genome was assembled using sequencing data generated from a single nematode to reduce the level of heterozygosity and facilitate assembly. First, long-read sequences were assembled using Flye (v 2.9-b1768 – Kolmogorov *et al*., 2019) in default setting. Second, short-reads were assembled using HASLR (v 0.8a1 – Haghshenas *et al*., 2020) assisted by long-read sequences. These assemblies were then merged using Quickmerge (v 0.3) prior to scaffolding using LINKS (v 1.8.7 – Warren *et al*., 2015) with Q20+ long reads derived from the pool of J3. This assembly was further scaffolded using information provided by RNA-seq with Rascaf (v 1.0.2 – Song *et al*., 2016). Finally, this scaffolded assembly was polished and corrected using five cycles of Pilon (v 1.23 – Walker *et al*., 2014) supplemented with single-nematode whole-genome sequencing data. The final corrected draft was analyzed for contaminating scaffolds with BlobToolKit (v 3.1.6 – Challis *et al*., 2020), and the quality and completeness of the genome were evaluated using BUSCO with the built-in Augustus gene predictor option set (v 5.3.2 – eukaryota_odb10 – Manni *et al*., 2021). The genome was then masked using RepeatMasker (v 4.1.2-p1 – Smit *et al*., 2015), annotated using BRAKER (Stanke *et al*., 2006, 2008; Li *et al*., 2009; Barnett *et al*., 2011; Lomsadze *et al*., 2014; Hoff *et al*., 2016, 2019; Bruna *et al*., 2021), retrained using the RNA-seq data. The draft genome size of *B. listronoti* was 80,637,718 bp assembled into 152 scaffolds, with an average G + C content of 33.08%, an L50 of 7 contigs, and an N50 of 4,373,361 bp. Completeness analysis using eukaryotic BUSCO genes yielded a score of 75.3% (C: 75.3% [S: 61.6%, D: 13.7%], F: 9.0%, M: 15.7%, n: 255). This is slightly lower than that obtained with the genome of *Deladenus siricidicola* (C: 87.1% [S: 86.3%, D: 0.8%], F: 5.5%, M: 7.4%, n: 255), a phylogenetically related species of insect-parasitic nematode (assembly accession GCA_009724625.1). Also, 13.7% of these BUSCO genes were found to be duplicated, indicating that a low level of haplotypic duplication may remain. This could also explain the small difference between the genome size (80.6 Mb) and its estimation by GenomeScope (69.8 Mb). BRAKER predicted 16,831 protein-coding genes. Functional annotation was accomplished by querying these predictions against the NCBI nr database using BLASTP (v. 2.6.0 – Altschul *et al*., 1990). This is the first genome release for *B. listronoti* and for any Allantonematidae species.

## Data availability and accession numbers

All the raw sequences and genome assembly files are available under the NCBI BioProject PRJNA842945. This whole-genome shotgun project has been deposited at DDBJ/ENA/GenBank under the accession JANLNO000000000.
